# Impact of Frailty on Functional Improvement Following Traumatic Spinal Cord Injury: A Japanese Single-Center Experience

**DOI:** 10.3390/jcm13144154

**Published:** 2024-07-16

**Authors:** Tsunehiko Konomi, Minako Yoshikawa, Keita Kajikawa, Takahiro Kitagawa, Yoshiomi Kobayashi, Mitsuru Furukawa, Kanehiro Fujiyoshi, Yoshiyuki Yato

**Affiliations:** 1 Department of Orthopaedic Surgery, Murayama Medical Center, National Hospital Organization, 2-37-1 Gakuen Musashimurayama, Tokyo 208-0011, Japan; 2 Nursing Department, Murayama Medical Center, National Hospital Organization, 2-37-1 Gakuen Musashimurayama, Tokyo 208-0011, Japan; 3 Department of Orthopaedic Surgery, National Defense Medical College, 3-2 Namiki Tokorozawa, Saitama 359-8513, Japan

**Keywords:** spinal cord injury, frailty, spinal cord independence measure

## Abstract

**Study Design**: This is a retrospective case series study. **Objective:** The aim of this study was to investigate whether frailty contributes to functional recovery in individuals with spinal cord injury (SCI). **Methods:** A total of 121 patients with SCI (106 cervical SCI, 15 thoracic SCI) discharged from our center over the past three years were studied. Moreover, 11-factor modified frailty index (mFI) scores, the length of hospital stays, the rate of returning home, and improvement in Spinal Cord Independence Measure (SCIM) scores were assessed retrospectively. **Results:** The average age at the time of injury for all 121 cases was 59.6 years. Based on pre-injury assessments, 24 cases were categorized as the Frail group, and 97 cases were categorized as the Robust group. The Frail group had SCIM improvement rates of 16.7% and a home discharge rate of 45.8%. In contrast, the Robust group had SCIM improvement rates of 33.5% and a home discharge rate of 68.0%, with statistically significant differences between the two groups. A significant negative correlation was observed between mFI scores and SCIM improvement rates (R = −0.231, *p* = 0.014). **Conclusions:** This study suggests that individuals with pre-existing frailty before SCI experience poorer SCIM improvement rates and face challenges in returning home.

## 1. Introduction

Japan is one of the world’s leading longevity countries, and the rapid increase in elderly individuals requiring care due to swift aging is becoming a serious issue. One of the issues associated with aging is the increase in elderly individuals requiring long-term care, which leads to rising costs for care and preventive services. In elderly individuals, physiological reserves gradually decline, leading to a loss of homeostasis. While there are cases where individuals suddenly transition from a healthy state to requiring care, such as in the event of a stroke, many elderly people gradually progress to a state of needing care through an intermediate stage known as “frailty” [1,2]. Frailty is characterized by increased vulnerability to stress due to the decline in physiological reserves associated with aging, resulting in outcomes such as functional impairment, the need for long-term care, and mortality. It encompasses not only physical issues such as decreased muscle strength, leading to reduced agility and increased risk of falls, but also psychological and social problems, including cognitive impairment, depression, living alone, and financial difficulties. In other words, frailty is defined as a state where the ability to adapt to stress diminishes due to a decline in reserve capacity associated with aging, making individuals susceptible to losing their social and physical independence and increasing the risk of requiring care [3].

In recent years, the importance of frailty has been highlighted not only from a pathophysiological perspective but also from a diagnostic and preventive care standpoint in the field of geriatric medicine. By raising awareness of the significance of frailty among both healthcare professionals and the public, advancements in preventive care can be achieved, leading to a reduction in the number of elderly individuals requiring care [4]. However, it should be noted that frailty is distinct from biological age, and not all elderly individuals necessarily become frail [5,6]. It is caused by various physiological and psychosocial factors associated with aging, including imbalances, muscle weakness, malnutrition, and chronic diseases. Frailty can impact daily activities and independence.

Spinal cord injury (SCI) occurs when there is damage to the spinal cord, and its symptoms and effects vary depending on the location and severity of the injury. SCI is often caused by accidents or sports injuries and can result in paralysis, sensory impairment, and the dysfunction of the urinary and bowel systems. It is considered a serious condition that requires rehabilitation and medical intervention. Acute medical care for individuals with SCI often requires extended hospitalization periods due to the time needed for rehabilitation and discharge planning, along with the prevalence of complications, making it challenging [7]. Furthermore, in Japan, there has been a significant increase in the aging population of individuals with SCI [8], and there is a growing number of elderly individuals sustaining minor trauma resulting in SCI [9].

In SCIs, there are cases where even though the paralysis appears similar during the acute phase immediately after the injury, some degree of recovery is observed over time (incomplete injury), and cases where no recovery is seen at all (complete injury). This largely depends on the extent of the damage to the spinal cord. Particularly in thoracic spinal injuries, complete injuries are more common, which is speculated to be due to the high-energy trauma often involved [10,11]. Additionally, it is said that if a complete injury remains unchanged after eight weeks post injury, the likelihood of any improvement in paralysis is minimal [12]. In complete injuries, the white matter structure of the spinal cord (the fibers and bundles of nerves transmitting signals from the brain and periphery) is severely damaged and sometimes even severed. On the other hand, in incomplete injuries, the white matter structure of the spinal cord is somewhat preserved, allowing for the reconstruction of remaining neural circuits through rehabilitation, leading to improvement in paralysis.

SCI and aging are closely related, and the following issues can be highlighted: First, there is the increasing risk of SCI among the elderly. As people age, their bone density decreases, and their physical flexibility and muscle strength decline, increasing the risk of SCI from falls or accidents. Additionally, the spinal column in elderly individuals is more fragile than in younger people, making them more susceptible to severe injuries from the same type of accident. According to Miyakoshi et al., the estimated annual incidence of traumatic SCI excluding Frankel grade E in Japan was 49 cases per million, with a median age of 70.0 years and individuals in their 70s being the most common age group [9]. The male-to-female ratio was 3:1. Cervical cord injuries accounted for 88.1% of cases. Following Frankel grade D as the most common grade (46.3%), Frankel grade C was the next most common (33.0%). The most frequent cause was falls on level surfaces (38.6%), followed by traffic accidents (20.1%). The proportion of falls on level surfaces increased with age. Second, there is the difficulty of rehabilitation. For elderly individuals, recovering from functional impairments caused by SCI and undergoing rehabilitation can be more challenging. As physical flexibility and muscle strength further decline, the existing health conditions often become more complicated, resulting in a prolonged rehabilitation process and making complete recovery difficult [13]. Third, there is the risk of complications. SCI in the elderly often occurs in conjunction with other health issues. For example, the motor function impairments caused by SCI increase the risk of other complications such as cardiovascular and respiratory diseases [14,15]. The clinical management of SCI in the elderly presents a significant challenge for clinicians. Fourth, there is the need for care and support. SCI in the elderly require the development of individualized care plans and support systems [7]. Due to the significant variation in physical limitations and loss of function among individuals, it is necessary to provide appropriate in-home support and medical care. Fifth, there are psychological impacts. In the case of elderly individuals, the physical limitations caused by SCI can lead to psychological effects. There is an increased risk of stress and depression due to the loss of independence and decline in quality of life [16].

Considering these points, SCI in the elderly manifests more complex issues, compounded by age-related physiological changes and health risks. Generally, in clinical decision making, the risk of poor prognosis is objectively assessed, taking into account the overall accumulation of deficits. However, SCI often involves underlying frailty, further complicating clinical decision making. Therefore, assessing the state of frailty in patients with SCI in advance may be beneficial for risk management.

The objective of this study is to examine the extent to which frailty contributes to the functional recovery of patients with SCI using the Spinal Cord Independence Measure (SCIM) [17,18] as an outcome measure. Through this investigation, we seek to clarify the interplay between SCI, aging, and frailty. This understanding will shed light on how frailty affects recovery outcomes post SCI, thereby enhancing the quality of care in SCI management.

## 2. Material and Methods

This study is a retrospective case series study, aiming to investigate the pre-injury frailty status, length of hospitalization, rate of discharge home, and SCIM improvement rate in 121 cases of patients with traumatic SCI discharged from Japanese single center during 3 years (from April 2018 to March 2021). Among these cases, 106 involved cervical spine injuries and 15 involved thoracic spine injuries. Frailty was assessed using the 11-factor modified frailty index (mFI) [19], which was created by Tsiouris et al., who conducted a comparison between the original Canadian Study of Health and Aging Frailty Index (CSHA-FI) with variables measured in the National Surgical Quality Improvement Program (NSQIP) dataset. Eleven preoperative clinical NSQIP variables were identified that matched some of the original 71 CSHA-FI variables. The 11 variables were a history of diabetes mellitus, functional status (not independent), chronic obstructive pulmonary disease (COPD), or pneumonia (PNA), congestive heart failure (CHF), prior myocardial infarction (MI), percutaneous coronary intervention (PCI), or stenting or angina, hypertension requiring medication, peripheral vascular disease (PVD), or ischemic rest pain, impaired sensorium, transient ischemic attack (TIA), or cerebrovascular accident (CVA) with neurological deficits. Functional status was defined as 1 [patient is independent requiring no assistance for activities of daily living (ADLs)], 2 (patient is partially dependent on another person for ADLs), or 3 (patient is totally dependent on another individual for ADLs), and 2 or 3 is defined as not independent. The score is calculated by dividing the number of variables by the total number assessed (n/11). We retrospectively assessed whether each item applied to the patients before the injury through interviews and calculated the mFI value for each subject (range: 0.0–1.0). An increase in the mFI score implies increased frailty. We set the frailty cutoff value at 0.18 based on the previous literature and classified the subjects into two groups, the Robust group (mFI < 0.18) and the Frail group (mFI ≥ 0.18), for comparative analysis [19,20,21,22].

The patients’ functional performance was assessed with the SCIM III questionnaire within the first week after admission to the rehabilitation department and within the last week before discharge from the department [18]. SCIM III items were scored according to actual direct observations of patient performance by expert trained nurses. When scoring items by direct observation was not practical (for example, bowel habits, voiding, or transfer issues regarding the wheelchair/ground), information was obtained from a staff member who had been observing the patient during routine care. The improvement rate was calculated using the following formula: improvement rate (%) = [(score at discharge − score at admission)/(100 − score at admission)] × 100 [23]. Additionally, we collected data on the American Spinal Cord Injury Association impairment scale (AIS) at admission, length of hospitalization, and discharge destination from medical records.

Statistical analyses were performed using EZR software version 1.68 (Saitama Medical Center, Jichi Medical University, Saitama, SA, Japan) [24]. For categorical variables, the chi-square test was conducted, while the Kruskal–Wallis test was used for continuous variables. Receiver-operating characteristic (ROC) analysis was employed to determine the mFI cutoff value for home discharge. Pearson’s correlation coefficient was used to determine the correlations between the SCIM improvement rate and mFI score. Statistical significance was defined as *p* < 0.05 for all analyses.

This study was approved by Murayama medical center’s Institutional Review Board, and informed consent was obtained from the research subjects using the opt-out method in the Murayama Medical Center. This study was conducted in compliance with the principles of the Declaration of Helsinki.

## 3. Results

The average age at the time of injury for all 121 cases was 59.6 years. Based on pre-injury interviews, 24 cases were classified as the Frail group (19.8%, mean age 71.8 years), and 97 cases were classified as the Robust group (80.2%, mean age 56.6 years). In the Frail group, the age at the time of injury was higher, and the proportion of frailty increased with older age. There were no significant differences between the two groups in terms of gender, type, or AIS of the injury (Table 1).

The SCIM scores at admission were 18.5 points in the Frail group and 27.6 points in the Robust group, with no statistically significant difference. However, the SCIM improvement rates were 16.7% in the Frail group and 33.5% in the Robust group, with a significant difference observed between the two groups (Table 2). The rates of home discharge and length of hospital stay were as follows: 45.8% and 224.7 days for the Frail group and 68.0% and 254.1 days for the Robust group, with a significant difference in the rate of home discharge between the two groups. There was no significant difference between the two groups in the percentage of individuals requiring full assistance at admission and discharge. Additionally, there were no significant differences between the two groups in the incidence of tracheostomy, pressure ulcer development, or in-hospital mortality.

**Figure 1:**
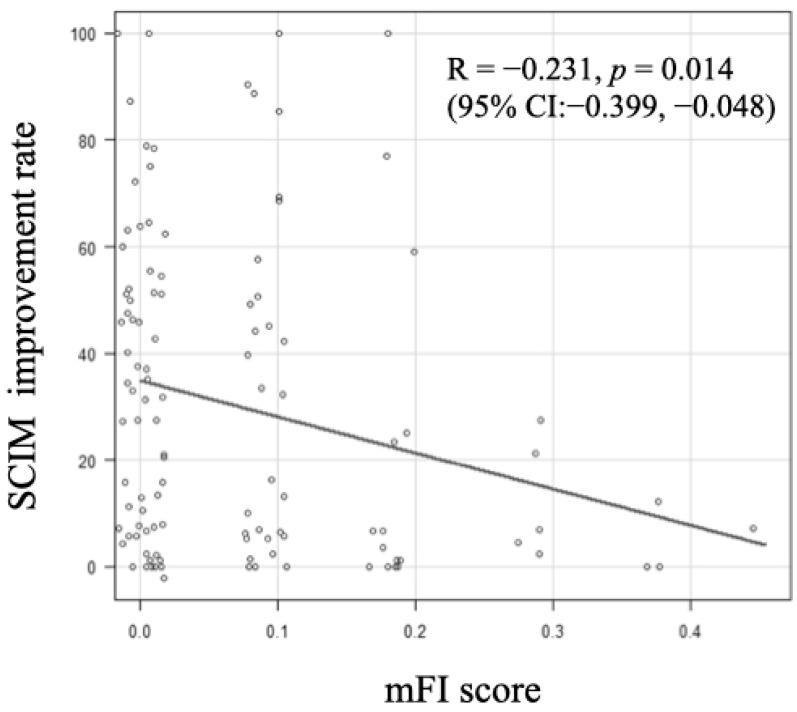
Association between modified frailty index (mFI) score and Spinal Cord Independence Measure (SCIM) improvement rate. When the value of mFI score was greater, the SCIM improvement rate decreased (R =−0.231, *p* = 0.014, 95% confidence interval (CI):−0.399,−0.048).

**Figure 2:**
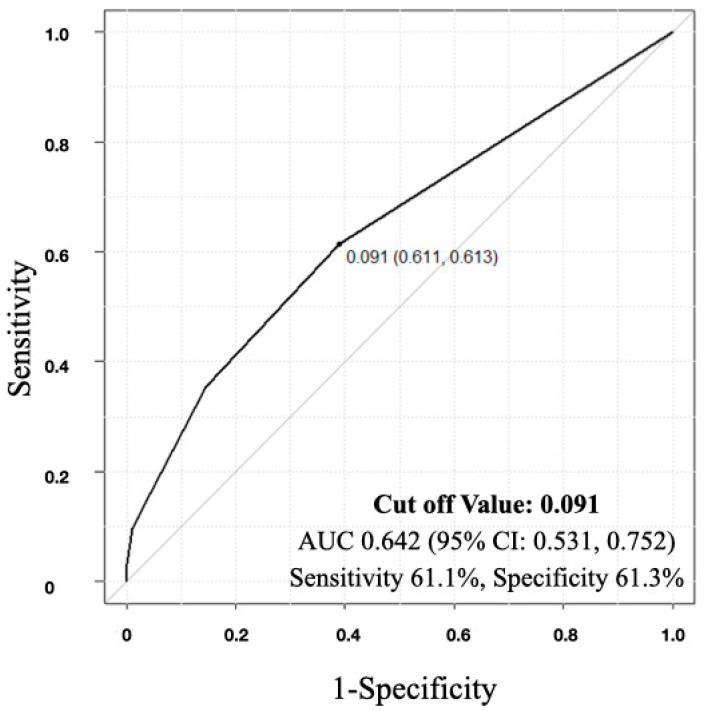
Receiver-operating characteristic curve indicating the cutoff value for modified frailty index (mFI) score in the detection of home discharge. The cutoff value was 0.091 in mFI score (area under the curve (AUC): 0.653, sensitivity: 61.1%, specificity: 61.3%, 95% confidence interval (CI): 0.531, 0.752).

A moderate but significant negative correlation was observed between the mFI score and the SCIM improvement rate, indicating that as the Frailty score increased, the SCIM improvement rate decreased (R = −0.231, *p* = 0.014, 95% confidence interval (CI): −0.399, −0.0482) (Figure 1). In the ROC analysis, the cutoff value of the mFI score that influenced home discharge was determined to be 0.091 (area under the curve (AUC): 0.642, 95% CI: 0.531, 0.752), with a sensitivity of 61.1% and specificity of 61.3% (Figure 2).

## 4. Discussion

In this study, we investigated the contribution of frailty to functional ADL recovery and discharge planning for patients with SCI using the SCIM as an evaluation tool for the first time to the best of our knowledge. The results indicated that the older the patients with SCI, the higher the proportion of individuals who were frail before their injury. When frailty was present, post-rehabilitation ADL function recovery was poorer, and there was a lower rate of returning home, suggesting a negative association between frailty and SCIM outcomes.

Frailty is caused by various factors, all of which are known to have significant impacts on individual patients [3]. Elderly individuals often experience physical weakness and muscle deterioration, leading to limitations in daily activities. This can make movement and self-care difficult, potentially resulting in the loss of independence. Additionally, frail elderly individuals tend to have poor balance, making them more prone to falls and fractures. This is due to the decline in physical function and muscle strength, posing an acute health risk. Moreover, frail elderly individuals may suffer from loss of appetite and malnutrition, leading to weight loss. Malnutrition can further weaken the immune system and worsen physical function, exacerbating their health condition [25]. Frailty is also closely linked to chronic diseases. For example, conditions like hypertension, diabetes, and cardiovascular diseases are risk factors for frailty, complicating the health status of the elderly. Psychosocial impacts are also observed in frail elderly individuals. They are more susceptible to psychological effects due to physical limitations and functional decline, experiencing loneliness, depression, and decreased self-esteem [26]. Furthermore, frailty can increase the need for medical and caregiving services, which can place a greater burden on families and caregivers, making the provision of care more challenging [7].

On the other hand, frailty diagnosis lacks a unified criterion, and numerous assessment methods have been proposed. Among these methods, two predominant approaches are commonly used [27]. One is the phenotype model (frailty phenotype), which evaluates aging-related signs (such as gait speed, muscle strength, and activity) in the assessment. It is often used for preoperative evaluations, investigating postoperative outcomes, and studying the prevalence of frailty among specific elderly populations. However, in cases like this study, where patients with SCI with limb paralysis or paraplegia are the subjects, it is not suitable to retrospectively evaluate physical function before the injury using the phenotype model.

The second approach is the deficit accumulation model (frailty index), which calculates the ratio of items that correspond to disability or functional decline based on a comprehensive functional assessment, diagnoses, and test results performed by the assessor. Therefore, it is applicable for frailty assessments regardless of the presence of physical disability. In studies involving patients with SCI, such as this one, the deficit accumulation model is more suitable for frailty evaluation. The mFI, based on the Canadian Study of Health and Aging Frailty Index, was created to detect perioperative risks in spinal surgery [21] and has been validated for its usefulness. However, the deficit accumulation model has its drawbacks, as it may not adequately account for physical function. In this study, we used the 11-factor modified frailty index. However, the usefulness of the 5-factor index has also been recognized, and it may be utilized in practice in the future [28].

In this study, the proportion of individuals diagnosed with frailty was 19.8% overall, and it reached 27.4% for those aged 60 or above. Similarly, Banaszek et al. reported that the prevalence of frailty diagnosis among patients with SCI was 2.8% for those under 60 and 30.6% for those aged 60 or above [29]. Furthermore, Chu et al. defined frailty as mFI-11 scores of 0.27 or higher and extracted data from a national sample of hospitalized patients in the United States (NIS) from 2016 to 2018. They found that 23.3% of patients with acute traumatic SCI aged 18 to 85 were classified as frail. Their study demonstrated an association between frailty and increased mortality rates, prolonged hospital stays, worsened discharge outcomes, and an increased risk of infections [30]. According to Dicpinigaitis et al., the proportion of frailty among surgically managed patients with traumatic SCI was 35.9%. Multivariate analysis results showed that frailty (mFI-11) was independently associated with a decreased likelihood of routine discharge and the occurrence of severe complications. However, they reported that frailty was not associated with in-hospital mortality or prolonged hospital stays [22]. Generally, the prevalence of frailty diagnosis among healthy individuals is reported to be 22.4% for those aged 65 or above, according to the Canadian National Population Health Survey [31]. Furthermore, more than one-third of individuals aged 85 or older are reported to be frail [3]. Considering that SCI in Japan are more likely to occur in elderly individuals due to falls on flat surfaces [9], it is suggested that older individuals with some form of frailty may be at risk of sustaining SCI, although there may be differences in evaluation methods and target populations between regions and ethnicities. Moreover, for patients with SCI with frailty, it has been reported that the length of hospitalization tends to be prolonged [29]. On the other hand, in older individuals with SCI, factors such as the motor score at admission and age itself are highly correlated with the occurrence of complications and in-hospital mortality, and the utility of frailty assessment is limited to patients with SCI under 60 years of age [29].

In this study, patients with SCI who were frail before the injury had a significantly lower rate of returning home (Frail group: 45.8% vs. Robust group: 68.0%) and a significantly lower ADL improvement in SCIM (Frail group: 16.7% vs. Robust group: 33.5%) compared to the control group. When the value of mFI score was greater, the SCIM improvement rate decreased (Figure 1). The average length of hospital stay for frail patients was 224.7 days, which was shorter than that of the robust group without significant differences observed. This is likely due to cases where patients were unable to return home and arrangements for facility admission progressed promptly as a result. Furthermore, there was no significant difference in the occurrence of complications and in-hospital mortality between the two groups. Although there was no significant difference in the SCIM score at admission between the two groups in this study, in general, lower initial SCIM scores and lower improvement rates make it more difficult for individuals to lead an independent life [32]. Therefore, assessing the frailty status when planning treatment may serve as a valuable reference indicator for predicting prognosis and discharge destination.

As a limitation of this study, the sample size was small, which resulted in a limited number of individuals diagnosed with frailty and posed challenges in interpreting the analysis results. Additionally, since this is a retrospective study, we cannot eliminate the influence of recall bias. Thus, it should be noted that the diagnostic criteria for frailty in this study were more stringent compared to previous reports. Due to the low proportion of frail individuals, we defined frailty as an mFI of 0.18 or higher for this analysis. Additionally, while ROC analysis was used to examine the correlation between mFI and returning home, an AUC of 0.642 suggests that the effectiveness of mFI could not be definitively proven based on this study alone. Furthermore, given that frailty rates tend to be higher among older individuals, analyzing the differences in the results between patients with SCI who exhibit frailty and those who do not within the elderly population, with a larger sample size, would be necessary.

For patients and their families, an SCI, whether complete or incomplete, is a personal experience. Even if we judge an injury to be minor, for the patient, even slight residual paralysis or disability can be a significant issue. It is the realistic role of medical professionals to help patients understand and accept their post-injury lifestyle, acquire the necessary skills, and move towards this new starting point together. This process still requires the involvement of many people, including family members and local staff, as well as considerable effort, medical resources, and time.

Considering the increasing aging population of patients with SCI in Japan, the results of this study suggest a potential association between frailty and SCI. To prevent an increase in SCI cases, nationwide efforts to prevent the development of frailty among elderly individuals will become increasingly important. Additionally, it is important to promote measures to prevent frail individuals from falling and sustaining SCI. Additionally, it is important to create a system that enables patients with SCI to return home.

## 5. Conclusions

The study suggests that as patients with SCI get older, the proportion of individuals who are frail before their injury is higher. Furthermore, if frailty is present, the functional recovery of ADLs after rehabilitation is poorer, and there is a lower rate of returning home. These issues highlight that SCI, aging, and frailty are complex health problems, and continuous care and preventive measures tailored to each individual’s condition are crucial. Preventing frailty and intervening early are essential for improving the health and quality of life of patients with SCI.

## Figures and Tables

**Table 1:** Background characteristics.

	Robust (*n* = 97)	Frail (*n* = 24)	*p*
Age at Injury (Year; Mean [SD])	56.6 ± 19.8	71.8 ± 9.7	<0.01
Age Demographic (n [%])			
<60	44 (45.4)	4 (16.7)	<0.05
60–74	34 (35.1)	11 (45.8)	-
≥75	19 (19.6)	9 (37.5)	-
Sex (Male/Female)	81:16	21:3	0.76
Injury Pattern (n [%])			
Paraplegia	13 (13.4)	2 (8.3)	0.73
Tetraplegia	84 (86.6)	22 (91.7)	-
Initial Neurological AIS Status (n [%])			
A	25 (28.7)	4 (19.0)	0.40
B	18 (20.7)	8 (38.1)	-
C	21 (24.1)	5 (23.8)	-
D	23 (26.4)	4 (19.0)	-

AIS: American Spinal Injury Association Impairment Scale.

**Table 2:** Summary of the patient cohort stratified by Robust vs. Frail.

	Robust (*n* = 97)	Frail (*n* = 24)	*p*
SCIM Score (Out of 100)			
Initial Score (Mean [SD])	27.6 ± 23.5	18.5 ± 16.7	0.09
Discharge Score (Mean [SD])	47.4 ± 29.9	30.7 ± 26.5	<0.05
Percent Recovery (%; Mean [SD])	33.5 ± 29.0	16.7 ± 26.7	<0.05
Hospital Stay (Day; Mean [SD])	254.1 ± 136.7	224.7 ± 129.8	0.34
Home Discharge (n [%])	66 (68.0)	11 (45.8)	<0.05
Requiring Total Assistance at Admission (n [%])	43 (45.3)	9 (37.5)	0.65
Requiring Total Assistance at Discharge (n [%])	20 (20.8)	7 (29.2)	0.42
Complication			
Tracheostomy (n [%])	16 (16.5)	4 (16.7)	1
Pressure Ulcer (n [%])	18 (18.6)	8 (33.3)	0.16
In-Hospital Death (n [%])	3 (3.1)	0 (0.0)	1

SCIM: Spinal Cord Independent Measure.

## Data Availability

Data are contained within the article.
